# Editorial: Assessment of osteoporotic fractures and risk prediction, volume II

**DOI:** 10.3389/fendo.2023.1238237

**Published:** 2023-07-14

**Authors:** Dandan Xie, ZhiFeng Sheng, Xiangbing Wang, Xiaoguang Cheng

**Affiliations:** ^1^Department of Clinical Nutrition, the First Affiliated Hospital of Hainan Medical University, Haikou, Hainan, China; ^2^Health Management Center, National Clinical Research Center for Metabolic Diseases, Hunan Provincial Key Laboratory of Metabolic Bone Diseases, Department of Metabolism and Endocrinology, The Second Xiangya Hospital of Central South University, Changsha, Hunan, China; ^3^Divisions of Endocrinology, Metabolism, and Nutrition, The State University of New Jersey, New Brunswick, NJ, United States; ^4^Department of Radiology, Beijing Jishuitan Hospital, Beijing, China

**Keywords:** osteoporosis, fracture, risk factors, prediction, assessment

With the progressive aging of the population, the prevalence of osteoporosis (OP) and associated fractures continues to rise, posing a significant global public health challenge. Recent reports have indicated that the annual incidence of osteoporotic fractures surpasses that of myocardial infarction, breast cancer, and prostate cancer combined (1). Hence, accurate prediction and early identification of individuals at risk of fractures are of utmost importance in mitigating osteoporotic fracture occurrences, improving patients’ quality of life and alleviating the burden on healthcare systems.

In our endeavor to gain deeper insights into the etiology, pathogenesis, diagnosis, treatment, epidemiological characteristics, and risk prediction of osteoporotic fractures, we organized a Research Topic that garnered an overwhelming response. The multitude of submissions received, especially those pertaining to early assessment of osteoporotic fractures, surpassed our initial expectations. Consequently, we have expanded this Research Topic into a two-volume collection to accommodate the significant number of high-quality submissions. In this summary, we present an overview of the contributions enclosed in the second volume.

In a series of contributions, multiple studies have focused on osteoporotic vertebral fractures (OVFs). Guo et al. conducted a study involving 2,874 postmenopausal women in Beijing, assessing four tools for identifying painful new OVF. Their findings revealed that the Fracture Risk Assessment Tool (FRAX) without bone mineral density (BMD) was the preferred option, while the Beijing Friendship Hospital Osteoporosis Screening Tool and Osteoporosis Self-Assessment Tool for Asians showed promise as simpler screening tools (Guo et al.). Another study developed and validated a deep learning model utilizing X-ray imaging data to enable artificial intelligence-based diagnosis and classification of vertebral compression fracture types. This technological advancement is expected to enhance the diagnostic accuracy of vertebral compression fractures in primary healthcare settings (Xu et al.). For patients with chronic OVF undergoing surgical treatment, Xin et al. reported that a scoring system based on five clinical characteristics—age, BMI, BMD, preoperative pelvic incidence-lumbar lordosis, and posterior ligamentous complex injury—exhibited good sensitivity and specificity in predicting the development of proximal junctional kyphosis after posterior internal fixation. Patients with a score of 6-11 were identified as being at high risk (Du et al.). In an analysis of patients treated with percutaneous vertebroplasty for compressive OVF, the authors identified BMD, bone cement disc leakage, and larger side bone cement volume/vertebral body volume ratio (LSBCV/VBV) as independent risk factors for postoperative adjacent vertebral compression fractures, with a significantly increased incidence observed when LSBCV/VBV reached 13.82% (Zhou et al.).

Regarding potential biomarkers of OP, N6-methyladenosine modulators have been useful as diagnostic biomarkers and for subtype identification in postmenopausal OP (Zhang et al.). Additionally, sclerostin has been identified as a potential biomarker for physical exercise in OP (Oniszczuk et al.). Another study reviewed the application of metabolomics in OP research (Zhao et al.). Regarding the prediction and screening of OP and fractures, Kong et al. identified chronic airway disease as a major risk factor for fractures in osteopenic women and proposed predictive models for major osteoporotic and hip fractures (Kong et al.). Furthermore, a small sample study initially compared the differences in vertebral mechanical properties estimated by finite element analysis with two computed tomography (CT) reconstruction kernels and evaluated their accuracy in the screening and classification of OP (Jiang et al.), which holds importance for the development of CT-based OP opportunistic screening tools. Two additional studies explored the association of hand grip strength and obstructive sleep apnea-hypopnea syndrome with BMD and fracture risk, respectively (Song et al., Wang et al.).

Moreover, within volume II; of this Research Topic, several studies have focused on fracture risk assessment in specific diseases. For type 2 diabetes mellitus (T2DM), one investigation demonstrated the utility of rheumatoid arthritis-adjusted FRAX as a valid clinical tool for evaluating fracture risk in postmenopausal T2DM patients, and a threshold of 4.156 mmol/L for advanced glycation end products was identified as a predictor of fracture risk (Gao et al.). However, the study found no significant association between metformin use and fracture risk in T2DM patients (Wang et al.). In another randomized study, the impact of antiretroviral therapy on bone quality in HIV-infected patients was investigated, switching from tenofovir disoproxil fumarate to tenofovir alafenamide for 24 weeks resulted in improved bone quality, independent of BMD (Soldado- Folgado et al.).

To summarize, this Research Topic provides significant insights into the screening, prediction, diagnosis, prognosis, and risk factors associated with BMD and fractures in OP, as well as disease-specific fracture risk studies. These findings, encompassing both molecular and clinical investigations, underscore the applicability of predictive tools and biomarkers for osteoporotic fractures while emphasizing the need to enhance the capacity of primary care institutions in identifying and diagnosing osteoporotic fractures. We believe that this Research Topic will contribute to the advancement of fracture prediction and identification in high-risk populations, ultimately reducing fracture incidence in clinical practice.

## Author contributions

DX, ZS, XW, and XC contributed to conception of the study. DX took responsibility for drafting the initial manuscript. All authors actively participated in manuscript revision, reading, and granting approval for the final submitted version.
